# Value-based approach to AVF maintenance: institutional micro-costing of drug-coated versus plain balloon angioplasty in a fixed-reimbursement system

**DOI:** 10.1186/s42155-026-00665-z

**Published:** 2026-02-28

**Authors:** Jernej Lučev, Dejan Dinevski, Robert Ekart, Silva Breznik

**Affiliations:** 1 Department of Radiology, University Medical Centre Maribor, Maribor, Slovenia; 2 Institute of Biomedical Informatics, Faculty of Medicine, University of Maribor, Maribor, Slovenia; 3 Clinic for Internal Medicine, Department of Dialysis, University Medical Centre Maribor, Maribor, Slovenia

**Keywords:** Arteriovenous fistula, Hemodialysis, Drug-coated balloon, Plain balloon angioplasty, Economic evaluation, Primary patency, Institutional policy, Value-based care

## Abstract

**Purpose:**

To evaluate the institutional cost-efficiency of paclitaxel drug-coated balloon (DCB; IN.PACT Admiral, Medtronic) versus plain balloon (PB) angioplasty for dysfunctional hemodialysis arteriovenous fistulas (AVFs) over 24 months, in order to inform resource allocation policy in a fixed-reimbursement system.

**Materials and methods:**

This analysis uses clinical outcomes from a previously published single-center cohort of 62 patients (31 DCB, 31 PB) treated for dysfunctional AVFs. The paclitaxel DCB used in the cohort was IN.PACT Admiral (Medtronic). A detailed institutional micro-costing approach based on cost-recovery self-pay tariffs was applied to quantify direct procedural costs. Total mean cost per patient and cost per year of primary patency (CPYPP) were calculated, and a sensitivity analysis was performed to explore the impact of varying the DCB device price.

**Results:**

The DCB group demonstrated higher mean target-lesion primary patency (1.46 ± 0.56 vs 0.86 ± 0.59 years) and required fewer AVF-related endovascular interventions per patient over 24 months (1.55 ± 0.81 vs 2.29 ± 0.94). Total mean cost per patient was lower with DCB (€8496.02 vs €11,324.55), resulting in a lower cost per year of primary patency (CPYPP €5819 vs €13,168). Sensitivity analysis suggested that this cost-saving profile remained robust across a wide range of DCB device prices.

**Conclusion:**

Despite the higher device cost, DCB angioplasty appeared to be a cost-saving and clinically more effective alternative to PB angioplasty in this cohort. Lower overall institutional expenditure and reduced reintervention frequency support its consideration for integration into AVF maintenance protocols and value-based care pathways in similar fixed-reimbursement settings.

## Introduction

Arteriovenous fistulas (AVFs) are recognized by major clinical guidelines, such as the KDOQI and EBPG, as the preferred vascular access modality for patients undergoing chronic hemodialysis (HD) due to superior long-term patency and lower morbidity rates compared to synthetic grafts and central venous catheters [1, 2]. Despite this gold-standard status, AVF dysfunction due to stenosis remains a critical clinical challenge, representing the most common cause of access failure and a leading source of hospitalization and healthcare expenditure within the HD population [3]. In the USA, vascular access-related complications contribute significantly to the total annual cost of dialysis care, exceeding billions of dollars [4].

The primary pathology driving AVF failure is neointimal hyperplasia (NIH), characterized by the pathological proliferation and migration of vascular smooth muscle cells (VSMCs) into the vessel intima [5]. This process is triggered by vessel wall injury from surgical anastomosis and repeated needle punctures, exacerbated by the turbulent blood flow and high shear stress environment characteristic of the AVF [6]. Conventional percutaneous transluminal angioplasty (PTA) using a plain balloon (PB) has long been the primary endovascular solution for stenosis [7]. However, the mechanical inflation required to open the lesion itself causes additional barotrauma to the vessel wall, accelerating the NIH cycle and resulting in restenosis rates that frequently necessitate multiple reinterventions within the first year [8, 9].

Drug-coated balloons (DCBs) were developed to interrupt this inflammatory-proliferative cycle. DCBs deliver a localized, therapeutic dose of an antiproliferative agent (paclitaxel) directly to the vessel wall during balloon inflation [9]. Paclitaxel acts by interfering with microtubule dynamics, effectively inhibiting the proliferation and migration of VSMCs, thereby slowing the progression of NIH and extending the duration of primary patency [10]. The success of this pharmacomechanical approach has been demonstrated in multiple clinical trials, including our own initial prospective study, confirming superior patency outcomes for DCBs in AVF interventions [11–13]. In parallel, sirolimus-coated balloon technology is being evaluated for dialysis access lesions, with early prospective data available [14].

The clinical superiority of DCBs is often challenged by their higher initial device cost. Health systems, particularly those operating under fixed or bundled reimbursement models, require robust economic data to determine if the clinical gain justifies the increased upfront expenditure [15, 16]. This is particularly relevant in systems where procedural reimbursement is fixed or bundled, making the cost-saving potential from avoided future procedures critical for budgetary approval. This study provides a rigorous, institution-specific economic evaluation comparing the DCB strategy to the PB strategy, utilizing micro-costing to derive a highly transparent and actionable institutional economic profile over a 24-month horizon.

## Materials and methods

### Study design, ethics, and patient data

This institutional economic evaluation (patency-based cost-effectiveness analysis) is a retrospective analysis based on prospectively collected clinical data from our previously published single-center study [11]. The paclitaxel DCB used in that cohort was IN.PACT Admiral (Medtronic, Minneapolis, MN, USA). That study included 62 consecutive adult patients with dysfunctional native AVFs who underwent angioplasty (31 in the PB group and 31 in the DCB group) and were followed for 24 months.

Baseline patient characteristics are summarized in Table 1. The original study found that the two groups were well matched across demographic variables like sex and age. However, a significant difference was noted in the median age of the AVF at the time of the index procedure, a factor that is discussed further in the limitations section.

**Table 1:** Baseline demographic and clinical characteristics

Characteristic	PB group (*n* = 31)	DCB group (*n* = 31)	*P*-value
Mean age (years)	67.03 ± 8.44	62.81 ± 17.2	0.22
Male gender	15 (48.4%)	16 (51.6%)	1
Hypertension	26 (83.9%)	18 (58.1%)	0.5
Hyperlipidemia	7 (22.6%)	5 (16.1%)	0.75
Diabetes mellitus	12 (38.7%)	13 (41.9%)	1
Smoking	5 (16.1%)	8 (25.8%)	0.53
AVF median age (days)	609 (294–991)	255 (178–465)	0.013
Radiocephalic fistula	20 (62.5%)	17 (54.8%)	0.6
Brachiocephalic fistula	8 (25.8%)	12 (38.7%)	0.4
Brachiobasilic fistula	3 (9.7%)	2 (6.6%)	1
Juxta-anastomotic stenosis	29 (93.5%)	30 (96.8%)	1
Arterial stenosis	1 (3.2%)	0 (0%)	1
Anastomotic stenosis	1 (3.2%)	1 (3.2%)	1
Stenosis median length (mm)	40 (40–60)	40 (40–80)	0.37
Technical success	31 (100%)	31 (100%)	1

Values are mean ± SD or median (IQR)

Group comparisons used Student’s *t* test or Mann–Whitney *U* test for continuous variables and chi-square or Fisher’s exact test for categorical variables

*PB* plain balloon, *DCB *drug-coated balloon, *AVF *arteriovenous fistula

The endovascular approach for the PB group consisted of standard angioplasty with a plain balloon, typically inflated for 60 s. The approach for the DCB group involved a sequential strategy: initial vessel preparation using a standard PB to achieve less than 30% residual stenosis, followed by definitive treatment with a paclitaxel DCB (IN.PACT Admiral). Pre-dilation PB inflation time was 60 s and DCB inflation time was 180 s, in accordance with the manufacturer’s instructions for use. Detailed procedural steps and lesion selection criteria are described in the index clinical study [11]. This technique aligns with international consensus guidelines which recommend adequate pre-dilation to optimize lesion expansion and promote effective drug transfer to the vessel wall [17].

### Clinical outcomes

The key clinical outcomes were taken directly from the published results of the index study [11]:

1. Mean number of AVF-related endovascular interventions per patient over 24 months: PB group = 2.29 ± 0.94 (median 2; IQR 2–3); DCB group = 1.55 ± 0.81 (median 1; IQR 1–2).
2. Mean Primary Patency (MPP) of the Index Procedure: Defined as the time from the index intervention to the next clinically indicated repeat intervention or thrombosis.
  ◦ PB group MPP: 315.7 ± 216.8 days (approx. 0.86 ± 0.59 years; median 0.74 years; IQR 0.42–1.05 years).
  ◦ DCB group MPP: 534.2 ± 206.1 days (approx. 1.46 ± 0.56 years; median 1.39 years; IQR 1.09–2.00 years).

### Economic analysis and perspective

The economic evaluation was conducted from the institutional / provider perspective over a 24-month time horizon. This perspective is vital for local administrators making resource allocation decisions. Costs were estimated using the hospital’s official self-pay price list, which allows for detailed cost capture. In our institution, these tariffs are derived from internal micro-costing and are designed to cover the full resource consumption of the procedure (no profit margin), making them a pragmatic proxy for true provider costs.

We employed a micro-costing (bottom-up) approach, which utilizes highly detailed, actual institutional costs (devices, labor, consumables, overhead) rather than aggregated national reimbursement rates (e.g., diagnosis-related groups or fixed tariffs) [18]. This was necessitated because the national health insurance system in our country operates on a fixed, flat-rate reimbursement model that does not account for the actual material consumption [19]. Using the self-pay list allowed for a superior estimation of the true resource consumption associated with each intervention, thereby avoiding the systematic biases inherent in "flat-rate" payment systems where actual material usage is often obscured [20]. Although absolute prices vary between institutions and countries, the relative difference between strategies in our model is driven by resource use (number of reinterventions) rather than by local pricing alone.

The direct procedural costs included the components detailed in Table 2. The standardized hospitalization cost was included to accurately reflect the institutional overhead, interventional suite time, nursing, and recovery resource burden associated with each procedure, irrespective of the patient's actual discharge status.

**Table 2:** Direct procedural cost per intervention by component

Component	PB procedure (€)	DCB procedure (€)
PB balloon (vessel preparation)	99.83	99.83
DCB balloon (device)	–	536.08
Labor cost per procedure	1942.51	1942.51
Additional material	536.00	536.00
Standardized hospitalization	2366.88	2366.88
Total cost per procedure	4945.22	5481.30

Costs reflect institutional cost-recovery micro-costing for a single procedure

*PB* plain balloon, *DCB* drug-coated balloon

### Calculation of economic metrics

1. Total mean cost per patient (TMC): Calculated as the mean number of interventions per patient multiplied by the total cost per procedure.
  $$\mathrm{TMC}\;=\;\mathrm{Avg}.\;\mathrm{interventions}\;\times\mathrm{Cost}\;\mathrm{per}\;\mathrm{procedure}$$
2. Cost per year of primary patency (CPYPP): Calculated as the total mean cost per patient over the 24-month follow-up divided by the mean primary patency achieved by the index procedure in years. This pragmatic, patency-normalized metric is conceptually analogous to “cost per day of patency” models used in vascular surgery to integrate durability and reintervention burden into economic assessment [21].
  $$\mathrm{CPYPP}\;=\;\mathrm{Total}\;\mathrm{mean}\;\mathrm{cost}\;\mathrm{per}\;\mathrm{patient}/\mathrm{Mean}\;\mathrm{primary}\;\mathrm{patency}\;(\mathrm{years})$$
3. Incremental cost-effectiveness ratio (ICER): Defined as the change in cost divided by the change in effect (ΔCost/ΔEffect). When a strategy achieves better outcomes at a lower cost, it is considered cost-saving (economically dominant) and explicit ICER calculation is not required [22].

## Results

### Clinical outcomes and procedure utilization

As previously published, the clinical effectiveness of DCB was superior, leading to a substantial reduction in the rate of reinterventions [11]. The DCB group required a mean of 1.55 interventions per patient over 24 months, compared to 2.29 in the PB group. The increase in mean primary patency was 0.60 years (approximately 70%) with the use of DCBs.

### Cost analysis

As detailed in Table 2, the cost per procedure was €4945.22 for the PB group and €5481.30 for the DCB group. The resulting total mean costs per patient are shown in Table 3.

**Table 3:** Total cost per patient over 24 months

Group	Avg. interventions	Cost per procedure (€)	Total cost per patient (€)
PB	2.29 ± 0.94	4945.22	11,324.55
DCB	1.55 ± 0.81	5481.30	8496.02

Reinterventions include any repeat percutaneous or surgical procedures performed to maintain access patency over 24 months

Avg. interventions are presented as mean ± standard deviation (SD) over the 24-month follow-up

*PB* plain balloon, *DCB *drug-coated balloon

Despite the higher device cost for the DCB strategy, the lower reintervention rate resulted in a significantly reduced total mean cost per patient over the 24-month follow-up period (€8496.02 vs €11,324.55). The total cost savings was €2828.53 per patient for the DCB strategy.

### Patency-based economic evaluation

The results demonstrate a pronounced difference in cost-efficiency when adjusted for clinical benefit (mean primary patency). The key metric, cost per year of primary patency (CPYPP), is presented in Table 4.

**Table 4:** Cost per year of primary patency (CPYPP)

Group	Total cost (€)	Patency (years)	CPYPP (€)
PB	11,324.55	0.86 ± 0.59	13,168
DCB	8,496.02	1.46 ± 0.56	5,819

CPYPP was calculated as the total mean cost per patient divided by the mean primary patency (years)

Patency values are presented as mean ± standard deviation (SD) in years over the 24-month horizon

*PB* plain balloon, *DCB *drug-coated balloon, *CPYPP *cost per year of primary patency

Given that the DCB strategy resulted in a lower total cost (cost difference: − €2828.53) and superior clinical outcome (patency difference: + 0.60 years), DCB angioplasty was both less costly and more effective than PB angioplasty in this cohort and can therefore be considered cost-saving from the institutional perspective.

### Sensitivity analysis: cost threshold for DCB

To test the robustness of this cost-saving finding, we performed a threshold analysis to determine the maximum DCB device price at which the DCB strategy would remain cost-saving (i.e., total DCB cost ≤ PB cost). A probabilistic sensitivity analysis was not undertaken because most cost inputs are deterministic fixed tariffs without institution-specific variance estimates; therefore, a threshold analysis provides the most transparent decision rule for local procurement planning.

The maximum sustainable DCB device price (*D*_max_) was calculated to be approximately €2360.94. Since the current DCB device price (€536.08) is substantially below this threshold, the DCB cost could increase by nearly 440% before the strategy would lose its absolute cost-saving advantage, strongly supporting the robustness of this cost-saving profile.

## Discussion

The findings of this study provide real-world evidence for the economic benefit of drug-coated balloon angioplasty in the management of dysfunctional AVFs. In our single-center cohort, the DCB strategy was associated with both superior mean primary patency and lower cumulative treatment costs over 24 months compared to PB angioplasty.

Importantly, although costs were sourced from the hospital self-pay tariff, this tariff in our institution is explicitly cost-recovery and is derived from internal micro-costing (staff time, suite use, hospitalization day, amortization, and consumables) rather than profit generation. It therefore approximates true provider costs for local decision-making. Nevertheless, absolute cost values will vary across hospitals and countries, and external generalizability is best interpreted in terms of relative resource use and the direction/magnitude of cost differences between strategies.

### Translating economic findings into clinical value

For interventionalists and hospital administrators, a strategy that is both less costly and more effective is of particular interest from a value-based care perspective. In economic terms, such a strategy can be described as cost-saving (economically dominant), although our results should be interpreted in light of the modest sample size and single-center design. In our cohort, the primary driver of the observed cost savings was the reduction in the frequency of reinterventions (from 2.29 to 1.55 per patient over 24 months). This reduction not only decreases total expenditure, but also increases intervention-free time for both patients and the interventional suite, potentially improving resource utilization.

### Operational efficiency and training impact

The observed reduction in reinterventions (0.74 fewer per patient over 24 months) is expected to translate into operational benefits. By dedicating fewer interventional suite hours to repeat, often routine, AVF procedures, resources (staff time, angiography equipment) may be freed up for more complex, urgent, or elective cases. This could improve scheduling flexibility and reduce bottlenecks, although workflow metrics were not directly measured in this study. A more stable workload may also support the training of junior interventional radiologists by reducing exposure to repetitive, low-yield reinterventions and allowing more time for critical diagnostic and therapeutic interventions [23].

### Clinical, quality of life, and financial implications

The nearly 56% reduction in the cost per year of primary patency (CPYPP) observed in our analysis indicates a substantial improvement in cost-efficiency with the DCB approach (Table 4).

Beyond the financial savings, reducing the number of interventions from 2.29 to 1.55 procedures per patient over 24 months lowers the procedural burden for patients and may positively influence quality of life, although QoL was not formally measured in this study. Each avoided intervention reduces procedural risk, limits exposure to contrast agents and radiation, and decreases time spent in the hospital or angiography suite. In addition, more stable access may reduce the need for temporary central venous catheters, which are associated with a higher risk of infection and morbidity [3].

### Addressing technical approach and patency

The success observed in the DCB group reinforces the importance of the sequential interventional approach (PB pre-dilation followed by DCB). This technique is designed to optimize the delivery of paclitaxel by ensuring full lesion expansion and maximal contact between the drug-coated surface and the arterial wall, facilitating optimal drug absorption into the media layer and enhancing the anti-proliferative effect [17].

### Contextualizing paclitaxel safety

It is critical for contemporary vascular interventional literature to address the widely discussed topic of paclitaxel safety, which arose following a meta-analysis suggesting a late-term mortality risk associated with paclitaxel-coated devices in the femoropopliteal segment [24]. While this concern predominantly relates to devices used in the peripheral arterial system, continuous long-term surveillance is necessary. Importantly, subsequent meta-analyses and dedicated studies in vascular access have generally demonstrated that this safety signal is not reproducible in the specific application of DCBs for AVFs, which operate under distinct hemodynamic conditions [12, 25]. Our clinical data align with the consensus that DCB use in AVFs is safe, and the economic benefits presented here must be weighed alongside the robust safety data specific to this anatomical application.

### Limitations and future directions

The primary limitation remains the single-center, non-randomized design, which introduces the potential for selection and institutional bias. Specifically, our original cohort showed a statistically significant difference in median AVF age between the groups (Table 1). While published data suggest that the age of a mature AVF is not a confounding factor for primary and secondary patency [26], we acknowledge this potential confounder. However, since the cost-saving effect is driven predominantly by the reduction in the number of reinterventions (a consequence of improved patency), rather than the absolute patency duration itself, the core economic finding remains robust.

In addition, the DCB protocol used a longer balloon inflation time (180 s) than the PB protocol (60 s). While prolonged inflation could be considered a potential procedural confounder, a prospective trial in femoropopliteal angioplasty found that prolonged dilation failed to improve long-term patency, suggesting that inflation time alone is unlikely to account for the observed durability differences [27]. This consideration was also discussed in the index clinical study [11].

Furthermore, this analysis only considers direct institutional costs. The exclusion of indirect and societal costs, such as patient transportation, lost work productivity, and caregiver burden, likely results in an underestimation of the total benefit of the DCB strategy. We did not perform a cost-utility analysis because the index clinical study did not collect patient-reported outcomes or utility weights required to estimate quality-adjusted life years (QALYs); additionally, isolating device-attributable QALY differences in this population would require dedicated longitudinal QoL instruments and modeling assumptions beyond the scope of a provider-focused micro-costing study. Future research should prioritize large-scale, multicenter randomized controlled trials (RCTs) with integrated, long-term economic endpoints, including QALYs and probabilistic sensitivity analyses, to provide a comprehensive societal evaluation of avoiding repeat procedures over time [3, 22].

### Sustainable impact on institutional policy

The finding that DCB was less costly and more effective in our setting supports a proactive discussion regarding its implications for institutional purchasing and procedural protocols. When comparing the return on investment (ROI) for a DCB versus a PB, the DCB offers immediate value creation through resource sparing. For interventional units, the reduced occupancy of angiography suites, decreased inventory turnover of disposable PB supplies, and maximized utilization of specialized staff hours translate into long-term systemic sustainability. The adoption of the DCB strategy moves vascular access maintenance from a reactive, repeated intervention model to a proactive, value-based care model, confirming that adopting higher-cost, high-performance technology may represent a fiscally responsible choice for modern healthcare providers facing similar constraints [2, 15].

## Conclusion

In this retrospective, single-center cohort within a fixed-tariff environment, DCB angioplasty was associated with lower mean institutional cost per patient (€8496.02 vs €11,324.55) and a lower patency-normalized cost (CPYPP: €5819 vs €13,168) than PB angioplasty over 24 months (Tables 3 and 4).

These findings suggest that, despite a higher device price, paclitaxel DCB angioplasty may be a cost-saving strategy for routine AVF maintenance in comparable fixed-reimbursement systems. Because costs and effectiveness can vary across institutions and across DCB platforms, confirmation in larger, multicenter studies and in different organizational contexts is warranted.

## Data Availability

The datasets generated and/or analyzed during the study are available from the corresponding author on reasonable request.
